# EXPANDABLE INTRAVERTEBRAL IMPLANTS IN POST-TRAUMATIC VERTEBRAL NECROSIS - NEW CLASSIFICATION SUGGESTION

**DOI:** 10.1590/1413-785220233104e262943

**Published:** 2023-07-31

**Authors:** DIOGO LINO MOURA, JOSUÉ PEREIRA GABRIEL

**Affiliations:** 1 Centro Hospitalar e Universitario de Coimbra, Serviço de Ortopedia, Setor de Coluna Vertebral, Coimbra, Portugal.; 2 Universidade de Coimbra, Faculdade de Medicina, Instituto de Anatomia e Clinica Universitaria de Ortopedia, Coimbra, Portugal.; 3 Grant Medical Center, Spine Institute of Ohio, Columbus, OH, United States.

**Keywords:** Necrosis, Pseudarthrosis, Spinal Fractures, Spine, Bone, Necrose, Pseudoartrose, Fraturas da Coluna Vertebral, Osso

## Abstract

The progressive evolution of post-traumatic vertebral necrosis and consequent loss of structural integrity of the vertebral body along with neurological risk, makes it one of the most feared and unpredictable pathologies in spine traumatology. Several studies have addressed the role of vertebroplasty, kyphoplasty, and corpectomy in its treatment; however, it remains a controversial concept without a defined therapeutic algorithm. The recent emergence of expandable intravertebral implants, which allow, by a percutaneous transpedicular application, the capacity for intrasomatic filling and maintenance of the height of the vertebral body, makes them a viable option, not only in the treatment of acute vertebral fractures, but also in non-union cases. In this study, we present a review of the current evidence on the application of expandable intravertebral implants in cases of post-traumatic vertebral necrosis. Based on the available scientific literature, including previous classifications of post-traumatic necrosis, and on the mechanical characteristics of the main expandable intravertebral implants currently available, we propose a simplified classification of this pathology, considering parameters that influence surgical therapeutic guidance, the morphology and the dynamics of the necrotic vertebra’s mobility. According to its stages and based on authors’ experience and on the scarce literature, we propose an initial therapeutic algorithm and suggest preventive strategies for this disease, considering its main risk factors, that is, fracture comminution and impairment of vertebral vascularity. Therefore, expandable intravertebral implants have a promising role in this condition; however, large prospective studies are needed to confirm their efficacy, to clarify the indications of each of these devices, and to validate the algorithm suggestion regarding treatment and prevention of post-traumatic vertebral necrosis. ***Level of Evidence III, Systematic Review/Actualization.***

## RESUMO

A evolução progressiva da necrose vertebral pós-traumática e consequente perda da integridade estrutural do corpo vertebral, juntamente com o risco neurológico, a torna uma das patologias mais temidas e imprevisíveis na traumatologia da coluna. Vários estudos têm abordado o papel da vertebroplastia, cifoplastia e corpectomia no tratamento da necrose vertebral, no entanto, o tratamento ainda é controverso sem um algoritmo terapêutico definido. O recente surgimento dos implantes intravertebrais expansíveis, que permitem através duma aplicaçao transpedicular percutânea a capacidade de preenchimento intrassomático e de manutenção da altura do corpo vertebral, torna-os uma opção viável não só no tratamento das fraturas vertebrais agudas, mas também em situações de não consolidação óssea. Neste estudo, apresentamos uma revisão das evidências atuais sobre a aplicação de implantes intravertebrais expansíveis em casos de necrose vertebral pós-traumática. Com base na literatura científica disponível, incluindo classificações prévias de necrose vertebral pós-traumática, e nas características mecânicas dos principais implantes intravertebrais expansíveis disponíveis, propomos uma classificação simplificada desta patologia, considerando parâmetros que influenciam a orientação terapêutica cirúrgica, a morfologia e a dinâmica da mobilidade da vértebra. De acordo com seus estágios e com base na experiência dos autores e na escassa literatura, propomos um algoritmo terapêutico inicial e sugerimos estratégias preventivas para esta doença, considerando seus principais fatores de risco, ou seja, cominução da fratura e lesão da vascularização vertebral. Portanto, os implantes intravertebrais expansíveis têm um papel promissor nessa condição; no entanto, estudos prospectivos de grande dimensão são necessários para confirmar sua eficácia, esclarecer as indicações de cada um desses dispositivos e validar a presente proposta do algoritmo de tratamento e prevenção da necrose vertebral pós-traumática. **Nível de Evidência III, Revisão Sistemática/Atualização.**

## INTRODUCTION

Post-traumatic osteonecrosis of the vertebral body was first reported in 1891 by Hermann Kummell, initially describing it as a vertebra collapse symptom that appears from weeks to months after a minor trauma, indicating the vertebral body’s nutritional insufficiency as the etiological hypothesis. ^(^ ^1^ ^),(^ ^2^ Initially, it was considered a rare condition; however, its diagnosis has been increasing, probably due to population aging, being more commonly found in the thoracolumbar transition and in older adults with osteoporosis. ^(^ ^3^ ^)-(^ ^7^ It is estimated that post-traumatic vertebral necrosis is underdiagnosed and that its real incidence is significant. Reports indicate a prevalence ranging from 7% to 37% of vertebral compression fractures, particularly affecting those with a more comminuted fracture pattern, greater flattening, and those occurring in less vascularized regions of the vertebral body, which are all known risk factors for the development of pseudarthrosis. A type of extrinsic interference has been described, consisting of an excessive load on a weakened fractured vertebra without enough stability to heal. Effectively, the vertebral fracture evolution to non-union bone, with progressive osteonecrosis of the vertebral body and the consequent loss of its structural integrity and neurological risk, is currently one of the most concerning and unpredictable challenges in spine traumatology. Currently accepted diagnostic criteria are patients who persist symptomatic from six weeks to three months after a vertebral fracture and patients who exhibit imaging signs of vertebral necrosis on computed tomography and magnetic resonance, with or without progressive flattening and collapse, or the development of intrasomatic clefts. ^(^ ^1^ ^),(^ ^2^ ^),(^ ^8^ ^)-(^ ^11^

Post-traumatic vertebral necrosis represents a failure in vertebral bone healing. Thus, it makes sense that the treatment aims to interrupt this disease evolution and negative consequences. This way, patients with symptomatic vertebral necrosis (axial pain and functional limitation), with or without nerve compression symptoms, are candidates for surgical intervention. Atrophic type pseudarthrosis in general fractures is usually treated with bone resection, repairing bony ends to restore blood and growth factors for the site; local application of bone graft, stimulating the process of bone healing; and fixation. However, in spine, cementoplasty techniques (vertebroplasty and kyphoplasty) have been used to treat this disease, immediately stabilizing the vertebral body without waiting for bone healing. ^(^ ^5^ ^)-(^ ^9^

Expandable intravertebral implants are self-expanding devices applied percutaneously with posterior transpedicular access. They are introduced inside the vertebral body and their expansion allows for restoring their height, integrity, and stability, when filled with bone cement or graft. The application of expandable intravertebral implants, sometimes referred to as armed kyphoplasty, in addition to allowing the immediate analgesia and stabilization benefits of vertebroplasty and kyphoplasty, can also creates a vertebral body metallic endoskeleton which ensures a greater strength and resistance and a long-term maintenance of restored vertebral height. This happens because vertebral endplates, after reduction, are mechanically supported by the expanded devices, decreasing or preventing vertebral flattening after its expansion and also lowering the risk of post-traumatic local and segmental kyphosis, in addition to ensuring very stable anterior support for the vertebral body. ^(^ ^12^ ^)-(^ ^26^ In Table 1, we present the characteristics of the two most commonly applied expandable intravertebral implants currently available: Vertebral Body Stenting (VBS^®^) and SpineJack^®^ systems. ^(^ ^12^ ^)-(^ ^26^ The evolution of the indications for these recent devices has also shown promising results in vertebral fractures which turn into chronic and symptomatic non-union situations. ^(^ ^18^ ^),(^ ^27^

**Table 1:** Biomechanical characteristics of the two most commonly applied expansive intravertebral implants currently available, the Vertebral Body Stenting^®^ and the SpineJack^®^.^12^ ^) (^ ^26^

Implant designation	Vertebral Body Stenting^®^	SpineJack^®^
**Illustration**	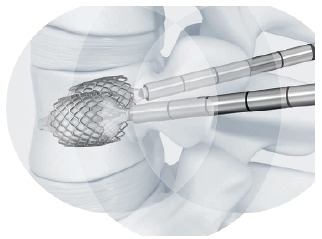	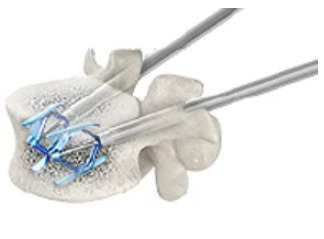
	http://synthes.vo.llnwd.net/o16/LLNWMB8/INT%20Mobile/Synthes%20International/SGT-EMEA-Agile/SE_818940AA/SE_818940AAeng.pdf	https://www.stryker.com/us/en/interventional-spine/products/spinejack-system.html
**Morphology**	Cylinder-shaped mesh (stent), two implants by transpedicular access	Similar to a car jack, with superior and inferior lamellas, and two implants by transpedicular access
**Material**	Chromium-cobalt	Titanium
**Expansion direction**	Centrifugal circumferential in the coronal plane (craniocaudal + lateral)	Bidirectional in craniocaudal or vertical direction
**Expansion mechanism**	Hydraulic mechanism, via a kyphoplasty balloon (controlled pressure and volume)	Mechanical mechanism
**Expansion power**	Maximum pressure = 30 Atm; Maximum expansion volumes: #small stent = 4 mL; #medium stent = 4.5 mL; #large stent = 5 mL	Expansion force = 500 Newtons; maximum expansion heights: #small implant 4.2 = 12.5 mm; #medium implant 5.0 = 17 mm; #large implant 5.8 = 20 mm
**Objective**	Vertebra reduction and space occupation	Vertebra reduction, preservation of unfractured trabeculae
**Rationale**	VBS^®^ is a reducing and space-occupying implant since it presents a multidirectional expansion (vertical and lateral). It is indicated for reconstruction or replacement of the vertebral body without the intention to wait for vertebral fracture natural healing. Stents are implants that, due to their expansion and impaction of the surrounding bone trabeculae, form two cavities inside the vertebral body, which are covered by an envelope of impacted trabeculae. These implants form cavities that, after being filled with bone cement or graft, replace a large part of the vertebral body, filling and stabilizing it. In addition, they minimize cement leakage by recreating the vertebral body walls by impacting bone trabeculae, thereby containing the cement inside	SpineJack^®^ is a more powerful reduction implant and preserver of unfractured native trabeculae. This implant is not as space occupant since it only expands vertically. In these cases, the goal is to reduce the fracture and wait for its healing, rather than replacing the vertebral body. This implant only reduces and supports the vertebral body, as it does not have a cavity shape or lateral expansion. Therefore, it does not destroy intact lateral trabeculae and does not create significant empty space within the vertebral body. Thus, this implant is useful in cases that demand fracture reduction and bone healing while preserving bone health. We consider that this implant is not ideal for replacing the comminuted, lytic, or porotic vertebral bodies with unstable interior content. Such cases require intrasomatic filling in addition to fracture reduction
**Cement Distribution Pattern**	Cavitary in the interior of the stents and trabecular at periphery	Trabecular, often joining the two implants in a horizontal pattern

## METHODS

**Figure 1:**
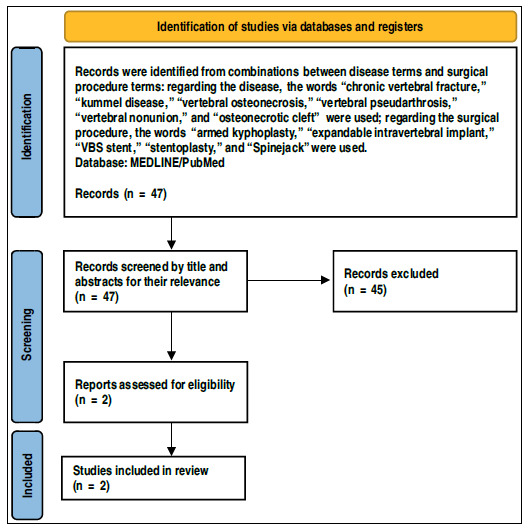
Preferred Reporting Items for Systematic Reviews and Meta-Analyses flow diagram. ^(^ ^28^

This study was based on a literature search in September 2021 on the MEDLINE/PubMed platform, with combination of terms concerning diagnosis and surgical procedure. The search terms for diagnostic words were “chronic vertebral fracture,” “kummel disease,” “vertebral osteonecrosis,” “vertebral pseudoarthrosis,” “vertebral nonunion,” and “osteonecroticcleft,” whereas search terms for surgical intervention were “armed kyphoplasty,” “expandable intravertebral implant,” “VBS stent,” “stentoplasty,” and “Spinejack”. A total of 47 results papers were found, of which, after reviewing titles and abstracts, only two were selected since they focused on the role of expandable intravertebral implants on post-traumatic necrosis or chronic fractures of thoracolumbar spine fractures (PRISMA chart in Figure 1). ^(^ ^18^ ^),(^ ^27^

## RESULTS

### Application of expandable intravertebral implants in post-traumatic vertebral necrosis - literature review

We selected an article on SpineJack^®^ expandable implants and another article on VBS^®^ stents, which are summarized in Table 2. Premat et al. ^(^ ^27^ analyzed the application of expandable intravertebral implants in chronic fractures, prospectively studying 19 consecutive adult patients who had undergone reduction and stabilization with SpineJack^®^ in Magerl A3 burst fractures, with the intervention occurring after a mean delay of 5.8 ± 2.9 months from the initial fracture. All consecutive adult patients with symptomatic osteoporotic vertebral compression fractures (OVCFs) who underwent vertebral augmentation with the SpineJack^®^ were prospectively included. Patients were considered eligible for inclusion if they met the following criteria: OVCFs involving the lower thoracic and/or lumbar vertebrae, considered unstable (grade A3 according to Magerl’s classification), kyphosis of at least 20° at the vertebra’s level, fractures older than six weeks, intractable back pain, with a visual analogue scale (VAS) of at least four. Preoperative evaluation included clinical examination and an imaging workup, including a computed tomography (CT) and a spine magnetic resonance imaging (MRI). All patients had postoperative spine and chest control X-rays in the operating room followed by a spine CT scan focused on the treated site. A systematic clinical follow-up was performed at one and six months after intervention. This way, they identified significant improvements between the preoperative visual analogue pain scale (median 7) and after six months postoperatively (median 2). The improvement in the mean local kyphosis was significant in 94.7% of the cases, going from 24.4 ± 4.1 to 11.7˚ ± 6.7. The mean Beck index increased significantly, from 0.43 to 0.66. Additionally, the anterior (11.2 mm ± 3.1 to 16.3 mm ± 2.7) and middle (11.5 mm ± 3.3 to 17.2 mm ± 2.7) vertebral heights also had significant improvements, with a smaller increase in posterior height (23.4 mm ± 3.4 to 24.2 mm ± 3.5). There was no worsening of posterior wall protrusions. The mean of injected cement was 5.9 ± 1.4 mL. By using computerized tomography (CT), the authors identified that 36.8% of patients presented discal extravasations, 15.4% presented venous extravasations, and one (5.3%) presented pulmonary embolism with cement, all asymptomatic. There were 21.1% of adjacent vertebral fractures, with a significant higher prevalence in cases with more accentuated corrections of the local kyphosis, which leads to the recommendation of prophylactic vertebroplasties at the adjacent levels in cases of chronic fractures with severe kyphosis.

**Table 2:** Current studies regarding the application of intravertebral implants in the context of chronic compression vertebral fractures. ^14^ ^)-(^ ^27^

Article	Premat et al. ^(^ ^27^	Distefano et al. ^(^ ^18^
**Nature**	Case series, prospective	Case series, retrospective
**Fracture type**	A3 compression chronic fracture (older than six weeks)	80 severe osteoporotic vertebral compression fractures - advanced collapse (Genant grade 3), high degree of osseous fragmentation (McCormack grade 2 and 3), burst morphology, pediculo-somatic junction fracture, and/or large osteonecrotic cleft.
**Number of fractured vertebrae**	19	Vertebrae with large osteonecrotic clefts in 56/80 levels (70%) of the sample
**Intervention**	Armed kyphoplasty with SpineJack^®^ implants	Stent-screw-assisted internal fixation (SAIF)
**Mean follow-up**	Six months	Six months
**Symptoms (VAS)**	Median VAS: 7 → 2 (p < 0.01)	VAS median 8 at preoperative → 3 at one month → 2 at six months (p < 0.05)
**Function**	57.9% of patients presented improvements in mobility, with nine patients (47.9%) able to fully ambulate without any help	PGIC Scale: 5.6 ± 0.9 at one month; 6.1 ± 0.9 at six months
**Imaging**	Parameters comparison from preoperative to postoperative: Mean kyphotic angulation: 24.4°± 4.1 → 11.7°± 6.7 (p < 0.01); Mean vertebral heights: anterior aspect 11.2 mm ± 3.1 → 16.3 mm ± 2.7(p < 0.01); middle aspect 11.5 mm ± 3.3 → 17.2 mm ± 2.7(p < 0.01); posterior aspect 23.4 mm ± 3.4 → 24.2 mm ± 3.5(p = 0.48); Modified Beck index 0.43 → 0.66 (p < 0.01)	Vertebral body reconstruction was evaluated by two external persons and considered satisfactory in 98.8% of levels, based on scores regarding correct placement and expansion of the implants, cement filling, and vertebral body height restoration.
**Complications**	21.1% of patients presented secondary adjacent level fractures correlated with kyphosis reduction; and 15.79% of patients presented minor PMMA	17.5% of patients presented painful adjacent vertebral fractures; 10% presented cement leakage detected on CT, with an epidural or foraminal location in 3.8%, all asymptomatic; 20.5% presented osseous subsidence around the VBS-cement complex, with mild to moderate secondary vertebral body height loss
**Conclusion**	Successful augmentation and reduction are reachable with SpineJack^®^ in chronic vertebral body fractures.	SAIF is a minimally invasive, safe, and effective treatment for severe osteoporotic vertebral compression fracture, including clefted vertebral bodies. VBS recreates the internal structure of the vertebral body, and favors a predictable and uniform cement distribution within the stents

VAS: Visual Analogue Scale; OSW: Oswestry disability score; PMMA: polymethylmethacrylate; Modified Beck index: minimal vertebral height/maximum vertebral height; PGIC Scale: Patient’s Global Impression of Change Scale; CT: computed tomography.

In the second included paper, Distefano et al. applied the stent-screw-assisted internal fixation (SAIF) technique, previously described by the same group, to treat 56 vertebrae with osteonecrotic intravertebral clefts. ^(^ ^18^ ^),(^ ^29^ The SAIF aims to complement the reduction and reconstruction of the vertebral body using VBS^®^ stents with pedicle screws, which anchor the stents-cement complex to the posterior elements. This method prevents their migration and acts as a bridge across the middle column, allowing union between the anterior and posterior portions of the vertebra, ensuring its integrity and preventing collapse and splitting. ^(^ ^29^ In another study by the same author, severe vertebral compression fractures were characterized by advanced collapse (Genant grade 3), a high degree of osseous fragmentation (McCormack grade 2 and 3), burst morphology with middle-column injury, pediculo-somatic junction fracture, and/or large osteonecrotic cleft, with several patients presenting more than one of these conditions. ^(^ ^18^ All patients underwent preprocedural spinal CT and/or MRI at the target level to accurately define the fracture morphology. Vertebral body reconstruction was assessed with post procedure radiographs and CT scan. Patients were followed-up at one and six months, with a clinical examination and upright plain radiographs. One of the problems involving the analysis of this article was the impossibility to isolate the results of vertebrae with necrotic clefts, since the authors do not separate the data by pathology groups; therefore, the study outcomes include acute comminuted fractures. Despite this, we consider that 70% of the sample with intravertebral clefts is a very relevant slice; thus, most cases correspond to situations of non-union or vertebral pseudarthrosis, so we present their results. Visual analog scale (VAS) scores improved with statistically significant difference from median 8 in preoperative to 3 at 1-month follow-up and to 2 at six months. The PGIC scale (Final Patients’ Global Impression of Change) was 5.6 ± 0.9 at one month and 6.1 ± 0.9 at six months, which indicates a positive subjective evaluation of their clinical improvement. There was a 17.5% rate of adjacent vertebral fractures, most of which were treated with vertebroplasty or SAIF. Cement leakage was detected in 10% of cases on post-procedure CT, with an epidural or foraminal location in 3.8% without any symptoms. Vertebral body reconstruction was evaluated by two external experts and considered satisfactory in 98.8% of levels, based on scores regarding correct placement and expansion of the implants, cement filling, and vertebral body height restoration. The authors highlight the importance of the SAIF technique in the stable reconstruction of the vertebra as a whole. They report that often in traditional vertebroplasty or kyphoplasty, only the anterior two thirds of the vertebral body are augmented for safety reasons to avoid intracanal cement leakage, turning the Denis’s middle column into a fragile ‘bare area.’ These areas favor bone reabsorption and refracture, cleavage, and splitting between the augmented anterior column and the middle column, with risk of posterior wall protrusion, focal kyphosis, instability, and neurologic injury. ^(^ ^18^ ^),(^ ^29^ Thus, they consider that, especially in unstable necrotic vertebrae-that is, with considerable intravertebral clefts, where the middle column is almost always affected-,traditional vertebroplasty and kyphoplasty may be insufficient since they do not strengthen this Denis column, which increases the risk of progressive bone resorption and vertebral collapse. The SAIF technique allows a 360° non-fusion interior vertebra reconstruction, in which stents restore the anterior column, whereas pedicle screws allow its anchorage to the posterior elements through the reinforcement of the middle column. ^(^ ^18^ ^),(^ ^29^ In short, both studies consider armed kyphoplasty with expandable intravertebral implants a successful minimally invasive option for the interior reconstruction of the vertebral body in non-union situations, obtaining excellent clinical and functional outcomes. Despite excellent outcomes, the first paper does not clarify what is the morphology and dynamics of the treated necrotic vertebra, whereas the second paper states that necrotic vertebrae were mobile, presenting large clefts. We think posttraumatic vertebral necrosis treatment and results should be analyzed separately according to a clear previous definition of the affected vertebra, clarifying vertebral necrosis presentation and stage and the performed treatment, mostly because the surgical options, its difficulties, and also their outcomes, are certainly distinct; therefore, as an example, the authors should refer to the following section of this study. Furthermore, about 20% of secondary adjacent level fractures seems to be a significant number and it is present in both studies; however, it is unclear whether it is a complication or a natural progression of osteoporotic spinal disease. Finally, the authors did not consider the severity of osteoporosis of each patient and the degree of correction of vertebral height that could justify prophylactic vertebroplasties at the adjacent levels.

## DISCUSSION

### Suggestion of therapeutic and preventive algorithm for post-traumatic vertebral necrosis

**Figure 2:**
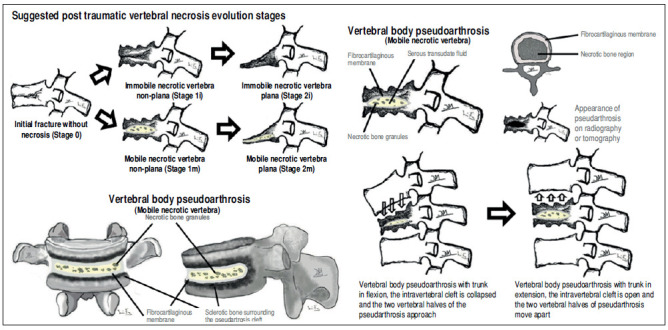
Suggested post-traumatic vertebral necrosis evolution stages: Stage 0 - Initial fracture without necrosis; Stage 1i - Immobile (i) necrotic vertebra non-plana; Stage 1m - Mobile (m) necrotic vertebra non-plana; Stage 2i - Immobile necrotic vertebra plana; Stage 2m - Mobile necrotic vertebra plana*;* Highlighting the presence of intravertebral cleft only in the mobile vertebrae. Immobile vertebrae do not present intravertebral cleft. On the right side, note the vertebral body pseudarthrosis or mobile necrotic vertebra morphology and biomechanics.

**Figure 3:**
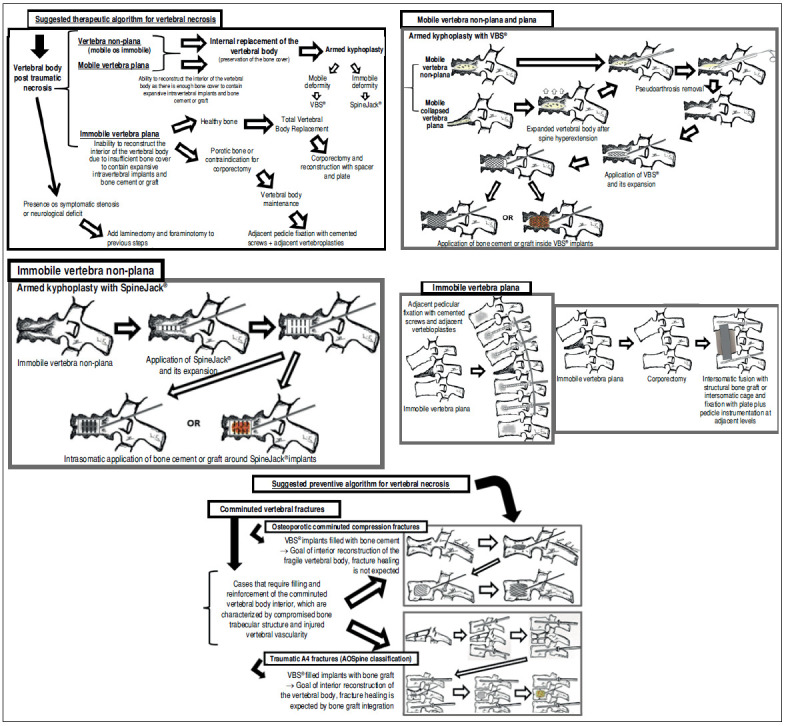
Suggested therapeutic and preventive algorithms for post-traumatic vertebral necrosis: Therapeutic algorithm for post-traumatic vertebral necrosis. Mobile vertebrae non-plana and plana - Armed kyphoplasty with VBS^®^. After removal of pseudarthrosis region (the same as the intravertebral cleft) and proper intravertebral cleaning, the implants are expanded and filled with bone cement or graft; Immobile vertebra non-plana - Armed kyphoplasty with SpineJack^®^. After proper intravertebral drilling, the implants are expanded and then bone cement or graft are applied around them; Immobile vertebra plana - the recommended treatment for young and active patients involves corpectomy and intersomatic fusion using a spacer (synthetic cage or structural allograft), along with fixation using a plate and pedicle instrumentation at adjacent levels. However, in older patients or cases where corpectomy is contraindicated, adjacent pedicular fixation with cemented screws and vertebroplasties at adjacent levels are indicated. Preventive algorithm for post-traumatic vertebral necrosis: 1 - For osteoporotic comminuted compression fractures, we recommend armed kyphoplasty with VBS^®^ filled with bone cement. In these patients the goal is interior replacement and reconstruction of the fragile vertebral body, fracture healing is not expected; 2 - For traumatic comminuted compression fractures (A4 from AOSpine classification), we recommend initial indirect reduction via adjacent pedicle instrumentation, followed by additional direct reduction and interior reconstruction of the vertebral body with VBS^®^ filled with cancellous bone graft. Highlighting the direct vertebral reduction that allows height restauration by elevation of the central depression of the upper vertebral endplate after expansion of VBS^®^ implants and their final filling with bone graft (yellow/brown final image representing the bone graft inside the stents).

Based on the scarce scientific literature available and on authors’ experience with expandable intravertebral implants, we propose an simplified classification for post-traumatic vertebral necrosis. This classification is based on parameters that directly influence the surgical therapeutic approach (Figure 2), namely the morphology and mobility dynamics of the necrotic vertebra (Figure 2). Furthermore, to aid in the management of this condition, we also propose a therapeutic and preventive algorithm for this disease (Figure 3). ^(^ ^30^ ^)-(^ ^35^ Therefore, we distinguish *two types of vertebral morphology*: *vertebra non-plana* and *vertebra plana*; *two types of mobility*: vertebrae with mobile deformity or in pseudarthrosis, characterized by intrasomatic clefts in the mobile region; and vertebrae with immobile deformity, that is, without evident intravertebral cleft. All these types of morphology and mobility can be combined in four stages, according to Figure 2. The determination of vertebral morphology and mobility in the context of post-traumatic necrosis must be performed by the combination of radiographs, including dynamic radiographs in hyperextension and orthostatism, computed tomography, and magnetic resonance imaging, also allowing to evaluate the amount of remaining bone tissue. The type of vertebral morphology and of the necrotic vertebra’s mobility will determine the surgical therapeutic option based on the possibility or not to preserve the vertebral body. ^(^ ^1^ ^)-(^ ^10^ ^),(^ ^36^

The authors define vertebra non-plana morphology as a vertebral body with a height that is equal to or greater than one third of the height of the original body along its entire length. We consider necrosis with vertebra non-plana to be a vertebral body still with sufficient bone tissue, namely with preserved bone cover (cortical ring and endplates), which allows for containing the application of expandable intravertebral implants, permitting a vertebral body interior reconstruction instead of its total replacement. Therefore, in these cases, we recommend armed kyphoplasty, in which empty spaces within the vertebral body are created by expandable intravertebral implants, which are surrounded by bone trabeculae impacted by the devices. Afterwards, the body is filled with bone cement or graft, which provides it with interior consistency and stability. In mobile vertebrae (pseudarthrosis), that is, with intravertebral clefts, regardless of their non-plana or plana morphology, it is possible to restore almost the entire height of the vertebral body by the positioning of the spine in hyperextension, which causes the separation of the upper and lower halves of the pseudarthrosis, increasing the cleft size and restoring the vertebral body height, which is filled internally. Thus, armed kyphoplasty is also indicated in these cases. The complete filling of the intrasomatic cleft is essential to stabilize the vertebral body, eliminating pathological intravertebral mobility. In turn, in vertebrae with immobile deformity, the goal is not to gain height, but only to fill the necrotic body, stabilizing it and preventing its progressive flattening by necrosis and bone resorption. ^(^ ^1^ ^)-(^ ^10^ ^),(^ ^36^

Given the lack of evidence in the current scientific literature on which expandable intravertebral implants to apply according to vertebral necrosis types and stages, the authors suggest, based mainly on clinical experience with the use of these devices and on treating this condition, in addition to current scientific evidence, an algorithm that considers vertebral morphology and mobility at each stage, as well as on the characteristics of each expandable device (Figure 3, Table 1). The present algorithm is not validated since literature is insufficient, so it should be seen as an initial suggestion of the role of expandable intravertebral implants in vertebral necrosis based on disease stage and progression, device characteristics, and personal experience of the authors. In vertebrae non-plana and in mobile vertebra plana, situations susceptible to armed kyphoplasty, we usually choose VBS^®^ implants in vertebrae with mobile deformity and SpineJack^®^ implants in those with immobile deformity (Figure 3). The VBS^®^ is an implant with a high capacity for space occupation, allowing the creation of large intrasomatic cavities with the cover made of the metallic mesh of the device and impacted bone trabeculae, which allows the application of a greater amount of bone cement or graft and, simultaneously, creates less pressure and more containment to minimize cement leakage. The cement filling in the VBS^®^ primarily follows a cavity pattern inside the stents. However, it also exhibits a trabecular pattern due to peripheral interdigitation, which establishes contact with an interior network of trabeculae-which penetrate the holes of the stent’s mesh upon expansion-and with the stent metallic network itself (Table 1). It is essential, in vertebral necrosis, for the cement agglomerate to be peripherally immobilized by the interdigitation in surrounding healthy bone trabeculae, which can only be achieved if there is an adequate previous removal of the fibrocartilaginous membrane and residues of pseudarthrosis, and of the peripheral sclerosis, minimizing the risk of cement and implant migration. Proper cleaning of the pseudarthrosis region, keeping only the bone cover of the vertebral body, is also essential when applying bone graft inside the stents, seeking to bring blood inside the vertebra. Thus, the necessary mediators are allowed to provide invasion by vessels of the bone graft matrix and osseointegration without interference from interposed necrotic tissues and fibrocartilaginous membrane, which characterizes the false joint and internally lines the intravertebral cleft, making local blood access difficult. ^(^ ^1^ ^)-(^ ^10^ ^),(^ ^13^ ^)-(^ ^18^ ^),(^ ^37^ ^),(^ ^38^

However, considering important sclerotic regions present in the vertebral body of the immobile deformity type and the hydraulic and pressure-dependent expansion mechanism of the VBS^®^, there is a risk that the resistance of the sclerotic bone is greater than the expandable capacity of these implants, and these may not expand or expand insufficiently, not creating the intrasomatic cavities of the desired size. Furthermore, in necrotic vertebrae with immobile deformity (without intravertebral cleft), vertebral expansion is not possible by positioning the spine in hyperextension; therefore, the creation of intrasomatic spaces is totally dependent on the action of intravertebral implants. Thus, in immobile vertebrae non-plana, we recommend SpineJack® implants, which, despite not being space-occupying implants, have a more powerful and mechanical expansion capacity, that is not directly dependent on pressure, managing to create intrasomatic spaces even in vertebrae with immobile deformity, which will be filled with bone cement or graft (Figure 3). The filling pattern of cement with SpineJack^®^ implants is mainly trabecular, as this implant only creates small cavities corresponding to its vertical expansion, so the cement, after occupying these small cavities, interdigitates in the surrounding trabecular space and often connects both implants in a horizontal pattern (Table 1). As previously mentioned, since SpineJack^®^ implants, unlike VBS^®^, do not create intrasomatic cavities that will contain the cement inside, their use in the context of vertebral necrosis-given the alternation of sclerotic with necrotic bone and the unpredictability of the vertebral body’s cortical ring-should imply a rigorous intraoperative fluoroscopic control when introducing bone cement to prevent its extravasation. ^(^ ^1^ ^),(^ ^19^ ^)-(^ ^26^ From a technical point of view, we highlight the probable difficulty in drilling and opening the interior of the vertebral body with immobile deformity, as it often alternates areas of very resistant sclerotic bone with fragile regions of necrotic bone, being necessary to be cautious in this gesture to avoid going beyond cortical walls and cause serious neurological and vascular damage.

The application of bone cement aims to fill and stabilize the interior of the vertebral body in an inert way, solving the problem of bone regeneration inability without waiting for bone healing. However, in post-traumatic vertebral necrosis in patients with young age and healthy bone, the authors defend that, instead of bone cement, the intrasomatic application of cancellous bone graft associated with expandable implants, seeking to obtain bone matrix colonization by osteoprogenitor cells, its vascular invasion and osseointegration, with the objective of achieving a vertebra that is biomechanically and physiologically more similar to the original in terms of loads distribution towards an active patient with a high functional demand in the future (Figure 4). We recommend the use of autologous cancellous graft extracted from the patient’s iliac bone for intrasomatic filling and, if the case demands more quantity, it is possible to mix the autograft with cancellous allograft from bone bank. In the same way of the treatment of general bone pseudarthrosis, in vertebral necrosis we sought to use a type of bone graft combining all the properties of osteoconduction, osteoinduction, osteointegration, and osteogenesis that are favorable to bone healing. ^(^ ^38^ ^)-(^ ^46^ The application of the bone graft combined with expandable intravertebral implants not only ensures the maintenance of vertebral height in time but also protects the bone graft from excessive loads, minimizing its damage and resorption until its osseointegration is achieved, allowing to obtain a totally bony vertebra with a metallic endoskeleton. The limited histological evidence conducted in cases without the use of intravertebral implants, has demonstrated, in some patients, the absence of intrasomatic graft integration, with frequent microscopic findings of partial graft necrosis even in the presence of clinical and imaging evidence of bone healing. This suggests a likely excessive load on the not yet osseointegrated graft (not protected by the intravertebral implant) and a weak histology-clinical correlation. Other studies have demonstrated the efficacy and revascularization of bone grafts applied in the context of vertebral pseudarthrosis. ^(^ ^38^ ^),(^ ^46^ ^)-(^ ^53^ However, long-term prospective studies are needed to demonstrate the advantage of intrasomatic application of bone graft associated with intravertebral implants in this context. As such, considering that functional age is more important than chronological age and that each patient must be considered individually, we empirically admit that, in individuals under 60 years of age, intrasomatic cancellous bone grafting should be preferred to bone cement. Over that age, the potential benefits of cancellous bone graft compared to bone cement filling become less evident, as such, in individuals older than 60 years of age, bone cement is usually applied. In short, the use of bilaterally expandable intravertebral implants and their symmetrical expansion allows a balanced filling of the vertebral body, providing the strength from the metal associated with the bone cement (simulates the concept of reinforced concrete from civil construction) and ensuring structural and protective support for its platforms until the intrasomatic bone graft is osseointegrated, restoring the body to its function of stable anterior support of loads and preventing its future flattening. ^(^ ^5^ ^),(^ ^8^ ^),(^ ^9^ ^),(^ ^13^ ^)-(^ ^26^ ^),(^ ^38^ ^),(^ ^46^ ^)-(^ ^53^

In turn, situations concerning the morphology of immobile vertebra plana, defined as those with a vertebral body with a height that is less than one third of the original on, in which there is no intravertebral cleft and the vertebral body bone tissue was practically completly reabsorbed, it is impossible to apply expandable intravertebral implants, as there is not enough somatic bone cover to allow a stable implant containment within vertebral bone tissue (Figures 2 and 3). Attempting to place expandable intravertebral implants in this type of vertebrae involves high risks and may have serious consequences, from migration of the implants, because they are not stable within bone tissue, with major neurological and vascular tissues injury risks, to important extravasation of cement or even inability to apply cement in the vertebra. As such, in cases of vertebra plana with immobile deformity, if the patient has conditions and functional expectations that justify it, the solution is the total replacement (exterior and interior) of the vertebral body through corpectomy and its replacement using a spacer (synthetic cage or structural allograft) with lateral plate fixation to adjacent vertebral bodies and pedicular instrumentation (Figure 3). However, these patients are often older adults, over 80 years old, presenting vertebrae with severe osteoporosis and various comorbidities. The patient’s own physiological condition may, by itself, contraindicate the invasiveness of the anterior approaches to abdominal or thoracic cavities, or an extensive posterior approach, needed for the corpectomy. The presence of porotic vertebrae increases the risk of adjacent vertebral fractures and loss of fixation in the intersomatic spacer after corpectomy. Therefore, in these cases, we recommend adjacent percutaneous pedicle fixation with cemented screws two levels above and below the level of the vertebra plana, to which we associate prophylactic vertebroplasties at the two adjacent upper and lower levels to the instrumentation, to minimize its overload and reduce junctional kyphosis and adjacent fracture (Figure 3). This treatment aims, by a less invasive treatment than corpectomy, to ensure for older patients a quick pain relief, as well as allowing early rise and walking. In sporadic cases of severe kyphosis in these osteoporotic patients with sagittal imbalance, Ponte osteotomies may be performed at some levels to minimize this deformity. ^(^ ^37^ ^),(^ ^42^ ^),(^ ^45^ ^),(^ ^47^ ^),(^ ^54^ ^)-(^ ^58^

Considering this algorithm, it is easily understood that we should early intervene in situations of post-traumatic vertebral necrosis, ideally in vertebrae non-plana stages (stages 1i and 1m - Figure 2), so that there is still enough bone tissue in the vertebral body to allow for the less invasive treatment, with percutaneous access and faster convalescence, the armed kyphoplasty. The most common evolution of vertebral necrosis is the progressive resorption of bone tissue; thus, we should not delay the indication of treatment with armed kyphoplasty. A late diagnosis or an unnecessary postponement of surgical intervention causes bone necrosis and resorption to progress, leading to situations of vertebra plana (stage 2) and increasing the risk of developing neurological damage due to posterior wall retropulsion and collapse of the vertebral body, which requires more aggressive surgical solutions.

Although there is no clear scientific evidence, the most probable and accepted cause of evolution of a vertebral fracture to non-union is the injury of intraosseous blood vessels during the fracture, compromising the vertebral body bone tissue blood supply, which prevents bone healing and favor progression to necrosis and pseudoarthrosis. ^(^ ^1^ ^)-(^ ^11^ However, up to the present day, there is no exam that allows to determine, in biological and vascular terms, that a given vertebra fracture pattern caused disruption of major intraosseous blood vessels and led to pseudarthrosis. As such, the authors consider that, in vertebral body comminuted fractures-those that reach the entire bone extension of the vertebral body, including both endplates and the posterior wall, which may be of traumatic origin (type A4 of the AOSpine classification^59^) or osteoporotic-, there is a high probability that the intraosseous vascularization of the vertebral body is compromised and will be insufficient to guarantee adequate bone healing. Thus, while the scientific literature has not evolved in determining the vascular biological importance within the treatment of thoracolumbar fractures, we exercise caution and, in fractures with high comminution (type A4 of the AOSpine classification^59^), we empirically consider that intraosseous vascularization is compromised, performing, as the fracture’s initial treatment, an immediate interior replacement of the vertebral body by an armed kyphoplasty with VBS^®^ expandable intravertebral implant filled with bone cement in osteoporotic fractures or with bone graft in traumatic fractures in individuals with healthy bone and under 60 years old (Figure 3). In type A4 traumatic fractures, we initially perform indirect reduction of the cortical ring and segment by ligamentotaxis and annulotaxis by maneuvers with pedicle screws in the adjacent vertebrae. Then, we perform additional direct reduction with VBS^®^ implants by multidirectional interior impaction of bone trabeculae, namely elevation of the central portion of the vertebral endplates, which guarantees anatomical reduction and its maintenance over time, as interior metallic supports (Figure 3). As for most osteoporotic compression fractures, usually without significant segmental kyphosis, isolated armed kyphoplasty is sufficient, without the need for adjacent pedicle instrumentation (Figure 3).

## CONCLUSION

This article reviews the promising role of expandable intravertebral implants in the treatment of post-traumatic vertebral necrosis and in its prevention in acute fractures with a high risk of non-union since these devices allow interior replacement of the vertebral body and stable anterior support of the spine by a percutaneous transpedicular approach. The authors propose a simplified classification of post-traumatic vertebral necrosis and a therapeutic algorithm based on the role of expandable intravertebral implants, reserving corpectomy or multilevel pedicle fixation only for immobile vertebrae plana. Currently, scientific evidence on the treatment of post-traumatic vertebral necrosis is limited, despite more studies have been addressing vertebroplasty and kyphoplasty more frequently, only a few focus on the application of intravertebral expansive implants in this context. Moreover, there is little scientific literature regarding the ability to identify high risk acute vertebral fractures that will evolve into non-union, thus enabling early action to prevent this dangerous disease. Large prospective studies are needed to clarify the indications for each of the expandable intravertebral implants in the treatment and prevention of post-traumatic vertebral necrosis and to consolidate their effectiveness.
